# Microstructural Changes of Aramid Fiber Due to Reaction with Toluene 2,4-diisocyanate under Tension in scCO_2_

**DOI:** 10.3390/polym11071110

**Published:** 2019-07-01

**Authors:** Haijuan Kong, Qian Xu, Muhuo Yu

**Affiliations:** 1 School of Materials Engineering, Shanghai University of Engineering Science, Shanghai 201620, China; 2 State Key Laboratory for Modification of Chemical Fibers and Polymer Materials, College of Material Science and Engineering, Donghua University, Shanghai 201620, China

**Keywords:** mechanical properties, tension, crystallinity, microfibril

## Abstract

High modulus aramid fiber, such as Kevlar 49, is conventionally prepared by the heat annealing of high strength aramid fiber under a suitable tension at high temperature, especially higher than 500 °C. This enables the mobility of a rigid molecule chain to be rearranged into a more perfect crystalline or orientation structure under tension. However, annealing decreases the tensile strength, since the thermal degradation of the molecular chain at high temperature cannot be avoided. Kevlar 49 fibers treated in supercritical carbon dioxide (scCO_2_) under tension could improve their mechanical properties at a low temperature. The effects of the tension on the mechanical properties and structure of the Kevlar 49 fibers were studied by mechanical testing, wide-angle and small-angle X-ray scattering (WAXS, SAXS), and scanning electron microscopy (SEM). The results show that the mechanical properties, crystallinity and orientation of the fiber can be improved when the tension is less than 0.6 cN/dtex, which may be due to the increasing of the mobility of a rigid segment with the help of the plasticization of scCO_2_ and re-arrangement of macromolecular chain into crystalline and orientation structure under tension. What’s more, the amorphous region also was enhanced by crosslinking reaction of toluene 2,4-diisocyanate (TDI) with the chain end groups of the macromolecules in the amorphous regions. However, a decrease of tenacity was found when the tension was higher than 0.6 cN/dtex, which is because the tension was so high that the microfibril was broken. The results indicated that treating the Kevlar 49 fiber in scCO_2_ under a suitable tension with TDI as a crosslink agent can simultaneously improve both the tenacity and modulus of the fiber.

## 1. Introduction

Poly(*p*-phenylene terephthalamide) (PPTA) fiber, prepared from a liquid-crystal system in solution due to the rigidity of the molecular chains, has a highly oriented molecular structure and a high elastic modulus, tenacity, and thermal stability [1]. Edmunds et al. discussed the effects of the manufacturing process on the crystalline, macromolecular, and fibrillar structure for the skin and core [1,2,3]. The domains are oriented along the direction of the processing flow and may link up to form a fibrillar structure [1,3,4]. Panar also observed the radially oriented pleated structure which superimposed onto a supramolecular fibrillar structure, where the intact fiber consisted of a large number of fibrils [3].

Further, the chain end was modeled by Morgan et al., where the chain ends in the skin are randomly arranged and become more bunched towards the core, resulting in periodic weak planes where the chain ends cluster [1,4,5]. Small-angle X-ray scattering (SAXS) is a technique that is ideally suited for the analysis of defects, such as microvoids and microfibrils. The structural nature of PPTA fibers is responsible for their mechanical properties. According to Panar, these defect bands could possibly include 50% of the molecule chain ends which are bridged by extended chains across this defect zone [3]. Mathur demonstrated that the interfibrillar adhesion of Kevlar 49 fibers could be improved by infiltrating an opened fibrillar network of single filaments with various polymeric resins [6]. A thermal drawing treatment followed by additional stretching at high temperatures can improve the modulus of the fibers [7,8]. The strong intra-and intermolecular bonding by the excellent alignment of microfibrils throughout the fiber enable Kevlar® fibers to achieve an elastic modulus and tensile strengths in the order of 100 and 3 GPa, respectively [9,10]. The alignment of microfibrils might be beneficial for increasing the tensile strength and elastic modulus [11,12,13,14].

Supercritical CO_2_ (scCO_2_) is an environmentally friendly solvent and blowing agent in a wide range of applications including polymerization [15,16], polymer purification [17] and foaming [2,10,18,19], coating applications, heating transfer [20], and powder formation [21], which attributed to its characteristics of chemical inertness, non-toxicity, near-zero surface tension, great penetration ability, moderate supercritical condition, and low cost [22]. ScCO_2_ has been demonstrated to be an environmentally friendly alternative in some applications owing to its penetrating and swelling effects. Molla proposed single step process with green scCO_2_ dyeing while simultaneously functionalization antimicrobial fabrics without environmental pollution [10]. Picchioni studied using scCO_2_ as reaction medium to synthesis high molecular weight polycaprolactone-g-glycidyl methacrylate (PCL-g-GMA) [15]. It has been introduced to modify the microstructure of a polymer, such as the crystal structure [21,23,24,25,26]. The microstructures of polyethylene terephthalate (PET) fibers treated in scCO_2_, which aids in dyeing and improves the crystallinity, were studied, and the rearrangement of polymer chains in the amorphous phase was induced by cold drawing and exposure to scCO_2_ [27,28]. Luo studied the surface modification of aramid fiber in scCO_2_ with glycidyl-POSS to improve the surface adhesion of aramid fiber/epoxy resin [29]. We reported a method the hot stretching and cyclic reaction of polyacrylonitrile (PAN) fibers in scCO_2_ by controlling the temperature and tension to improve the mechanical properties or reduced the heat of the cyclic reaction [20,30]. In our earlier study, we reported a new method improving the mechanical properties of single aramid filament by forming a crosslinking network structure in the amorphous phase of a PPTA fiber with the aid of scCO_2_, which enhanced the amorphous phase and improved the tensile strength and modulus of the fiber [31]. However, the effects of the tension on the mechanical properties and microstructure have not been researched in detail.

The scCO_2_ can diffuse into the amorphous region of Kevlar 49 fiber to enable the mobility of rigid segment of macromolecular chain at a lower temperature, which can avoid thermal degradation at high temperature. When tension was applied, the segment chain can be rearranged into a regular structure, which could improve the mechanical properties. In this paper, the influence of the tension applied in scCO_2_ on the mechanical properties and microstructure of Kevlar 49 fibers was studied.

## 2. Experimental

### 2.1. Materials

Kevlar 49 fibers were manufactured by Dupont Co. USA (Dupont, Midland, MI, USA). The yarn is made of 10,012 single filaments with a diameter of 12.7 μm and a specific linear density of 6320 dtex, density of 1.44 g/cm^3^. The carbon dioxide, toluene 2,4-diisocyanate (TDI), and acetone used in this study were purchased from Shanghai Chenggong Gases Co., Ltd. (Shanghai, China), J&K Chemical Ltd. (Shanghai, China), and Shanghai Ling Feng Chemical Reagent Co., Ltd. (Shanghai, China), respectively. These chemicals were used without further purification.

### 2.2. Treatment in scCO_2_

Experiments were conducted using scCO_2_ with TDI, and the process is reported in our early study [32]. After being cleaned with acetone, the Kevlar 49 fibers were rolled on a stainless-steel formwork under different controlled tensions (0.2, 0.4, 0.6 and 0.8 cN/dtex). 15 wt% TDI, relative to the weight of the fibers, was firstly placed on a glass filter at the bottom of the reactor. When the vessel reached the set temperature of 120 °C, carbon dioxide gas was supplied via a high-pressure syringe pump and maintained at a preset pressure, 13 MPa. This pressure was maintained throughout the experiment. Subsequently, the system was kept stable for a certain period of time (120 min) to allow the TDI to dissolve in ScCO_2_ and react with the Kevlar 49 fibers. Finally, decompression was slowly conducted. The fibers treated were cleaned with acetone and dried under vacuum at 80 °C for 4 h.

### 2.3. Tensile Testing

The tensile strength and modulus of Kevlar 49 fibers as received and treated were tested by XQ-1A tensile tester (Shanghai New Fiber Instrument Co.,Ltd, Shanghai, China) with a gauge length of 10 mm and an extension rate of 10 mm·min^−1^. At least 50 specimens were tested for each sample, and the average values of the tenacity and modulus were calculated. The linear density of fiber was measured by XD-1 vibrating fiber fineness instrument. The unit conversion of Pa and CN/dtex was based on the Equation (1)

(1) $$\sigma =9.53\times 10^{7}\times \rho \times \frac{CN}{dtex}\;\left(pa\right)$$

where σ is the tensile strength of Pa unit, ρ is the density of the fiber as 1.44 g/cm^3^, *dtex* is the average linear density for 10 thousands of meters fibers.

### 2.4. Scanning Electron Microscopy

A JSM-5600 LV scanning electron microscope was employed to analyze the surface morphology of fiber which was etched by 85% H_2_SO_4_ for 20 s in order to remove the amorphous region. All the samples were coated with a vapor-deposited thin conducting layer of gold to minimize charging.

### 2.5. Thermogravimetric Analysis

A DuPont TGA-29 thermogravimetric analyzer (Dupont, USA) was used to measure the thermal stability of the as-received and treated fibers in scCO_2_. Temperature ramp measurements were conducted in an inert atmosphere of N_2_ from 30 to 900 °C at 10 °C·min^−1^.

### 2.6. WAXS and SAXS Measurements

WAXS experiments of the fibers were carried out at the SSRF Beamline 16 BL with an X-ray wavelength of 0.124 nm. Small bundles of fibers were vertically mounted onto a sample holder at a sample-to-detector (Mar CCD 165, Shanghai Synchrotron Radiation Facility, Shanghai, China) distance of 120.5 mm.

SAXS experiments of the fibers were carried out at the SSRF Beamline 16 BL with an X-ray wavelength of 0.124 nm. Small bundles of fibers were vertically mounted onto a sample holder at a sample-to-detector (Mar CCD 165, Shanghai Synchrotron Radiation Facility, Shanghai, China) distance of 5027.5 mm and calibrated with a chicken collagen standard.

### 2.7. WAXS and SAXS Data Analyses

**Figure 1:**
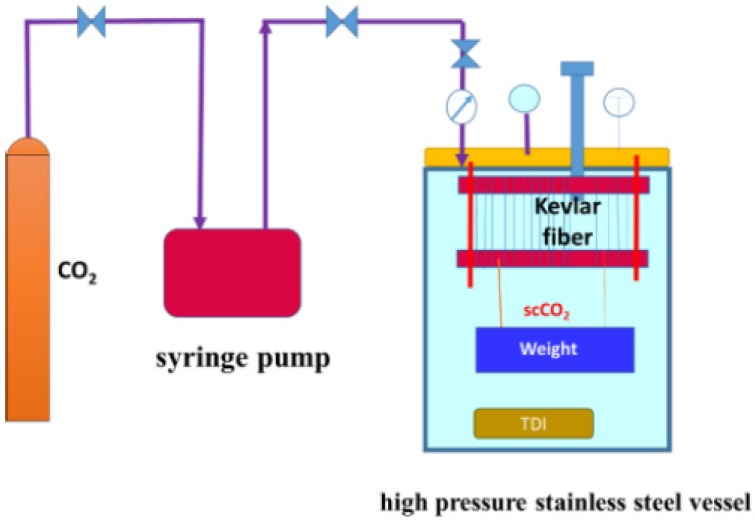
Schematic of experimental setup for treating Kevlar 49 fiber in scCO_2_.

**Figure 2:**
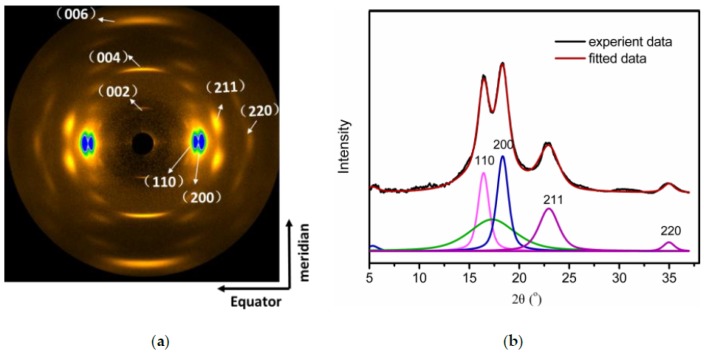
(**a**) Two dimensional wide-angle X-ray scattering (WAXS) patterns and (**b**) one-dimensional WAXS profiles and fitting results for Kevlar49 fibers as received.

The WAXS and SAXS data analyses were carried out using the Xpolar software (Stonybrook Technology and Applied Research, Inc., Stony Brook, NY, USA). The WAXS pattern of the as-received Kevlar fibers is shown in Figure 1a. Some sharp diffraction spots on the equator and streak-like layer were observed, which indicates that the (110), (200), and (211) reflections are located on the equator, while along the (002), (004), and (006) planes, meridional reflections are observed [33]. The one-dimensional WAXS plots along the azimuthal direction and fitted results for Kevlar49 fibers obtained from the 2D WAXS patterns as shown in Figure 2b. The 1D integrated intensity profiles were deconvoluted into crystalline and amorphous peaks. In the deconvolution process, three crystalline peaks ((110), (200), (211), and one amorphous peak were used. The results for determining the crystallinity and crystal size from the WAXS data are obtained from the fitted results.

Moreover, the amorphous halo along the equatorial direction indicates the poor intermolecular packing structure of chain segments parallel to the fiber axis.

The pattern shape on the equator was sharp along the azimuthal direction. Therefore, the alignment of molecules in the Kevlar49 fiber is parallel to the fiber direction. The bright area will be discussed later, it indicates crystal growth and orientation improvement.

The (200) and (110) diffractions were used to study the crystallites and size of the Kevlar crystal. Excellent alignment of the (200) lattice fringes, which represent the alignment of Kevlar molecules with respect to the fiber axis, was observed. The degree of orientation of the (200) lattice plane was calculated by Herman’s orientation function, as shown in Equation (2) [34]:

(2) $$f=\frac{3{cos}^{2}\varphi -1}{2}$$

where *cos*^2^*φ* is the average angle that chains make with the direction, which is the average direction of the orientation of the chains.

The crystallite sizes (ACS) in the directions perpendicular to of (110) and (200) crystal planes were determined by the Sherrer Equation (3) [35]

(3) $$L_{hkl}=\frac{0.9\times \lambda }{\beta \cos\theta }$$

where *λ* is the wave length of the X-ray source (0.124 nm) and *β* is the full width at the half maximum of the fitted scattering peak at the Bragg angle *θ* corresponding to the crystal plane (110) and (200).

Prior to the data analysis, the background scattering was corrected for by subtracting an average 2 s blank image from the sample scattering. Then, these data files were transformed into two-dimensional (2D) data. All data analyses were carried out using the X polar software. A SAXS pattern provides the average cross-sectional size of a cylindrical material that has a scattering power. It is difficult to determine whether the streak-like SAXS pattern originates from the microfibrils, microvoids, or reflections from the fiber surface depending on the angular range. Grubb et al. pointed out that the scattering objects in Kevlar 49 were mainly associated with the microfibrillar structure and not from the void morphology [36]. Dobb et al. also concluded that Kevlar exhibited no obvious voids [11]. Ran et al. were in agreement with the above, and their studies concluded that in Kevlar 49 fibers soaked with solvents of different densities, the shape and intensity of the streak patterns were always approximately the same [34]. For these reasons, the results of the present study are in agreement with these findings. Here, we analyze the equatorial streak in the SAXS pattern to study the microfibrils of Kevlar 49 fibers treated under different conditions. If the microfibrils are perfectly aligned along the fiber direction and have a finite length of *L*, then the width of the streak (*B_obs_*) in the reciprocal space is independent of the scattering vector (*s* = 2sinθ/*λ*, where 2θ is the scattering angle, and *λ* is the wavelength). The effects of both the finite length and orientation can contribute to the width of the equatorial scattering streak. The distribution of the scattering vector can be determined using the Ruland method [8,37].

**Figure 3:**
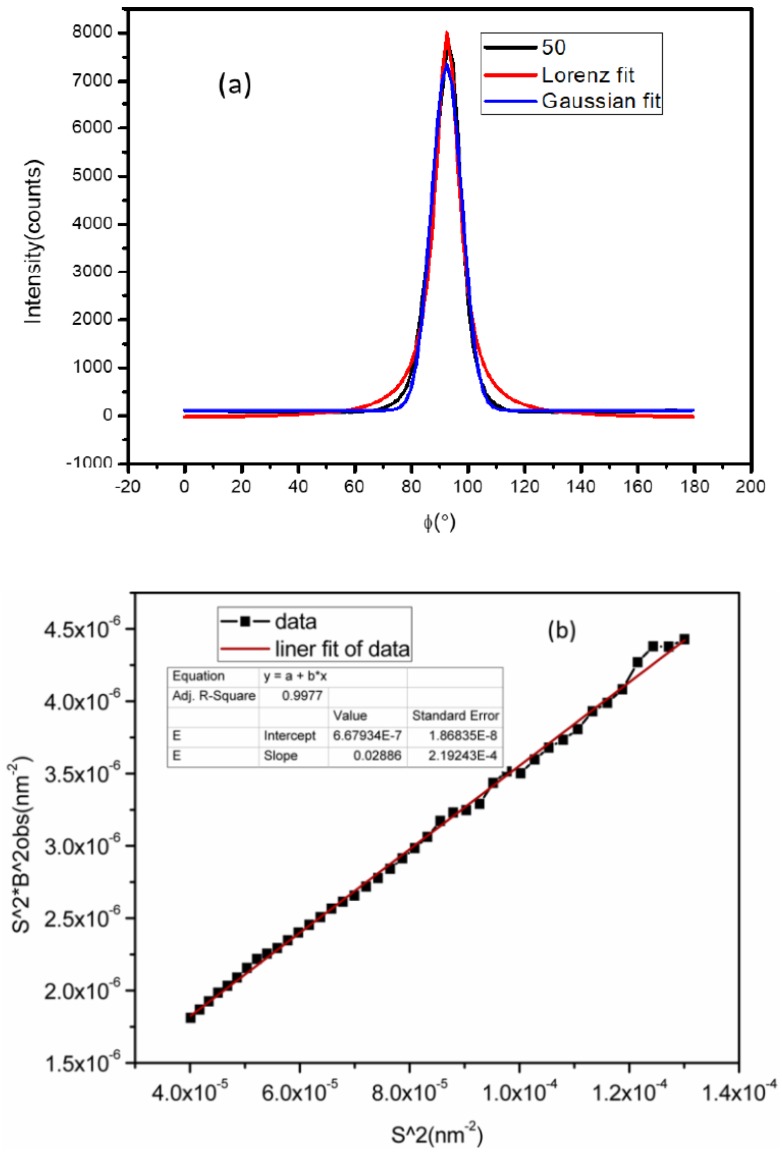
Representative azimuthal scans of the equatorial streak from the Kevlar49 Fibers (**a**) and the plots $s^{2}B_{obs}^{2}$ vs. *s*^2^ for Kevlar 49 fiber (**b**).

In Figure 3, an example of such an intensity distribution is shown as a function of the angle ϕ with respect to the principal axis. The azimuthal angle is approximately fitted with a Gaussian distribution rather than Lorentz distribution to estimate the full width at half maximum *B*_obs_. The microfibril length (*L_f_*) and disorientation width (*B*) can be obtained using Equation (4)

(4) $$s^{2}B_{obs}^{2}=\frac{1}{L_{f}^{2}}+s^{2}B_{\emptyset }^{2}$$

The relationship between $s^{2}B_{obs}^{2}$ and *S*^2^ of the as-received Kevlar49 fibers is linear, as shown in Figure 3b. To determine *L_f_* and *B_obs_*, the data is fitted using a linear least-squares fitting routine. The intercept at *s* = 0 determines the length of the microfibril, and the slope is related to the microfibril disorientation width.

## 3. Results and Discussion

### 3.1. The Amorphous Phase of the Kevlar 49 Fiber

**Figure 4:**
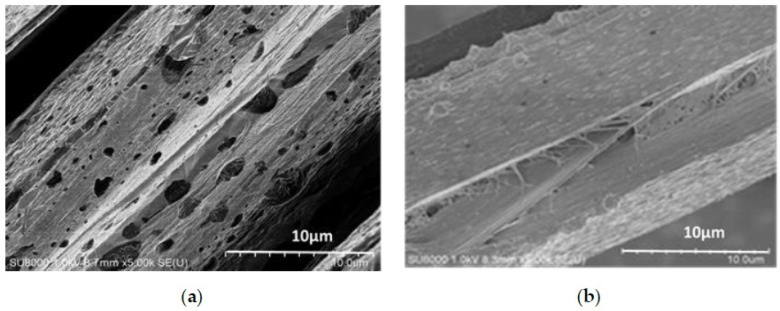
SEM images of the Kevlar 49 fibers etched by 85% H_2_SO_4_ (**a**) as received (**b**) treated in scCO_2_ with TDI under tension.

Figure 4 shows scanning electron micrographs of the fibers etched by 85% H_2_SO_4_ for 20 s; some fibrils running parallel to the fiber axis are also observed. Chemical etching techniques have been used to preferentially attack the amorphous regions of the fiber that have a higher chemical activity [38]. These regions are chain ends, misalignments perpendicular to the fiber axis, or residual stresses caused by crystalline imperfections. Figure 4b showed less void for fiber treated under tension with TDI in scCO_2_, compared with Figure 4a for the as-received fibers, which suggests that the amorphous phase is enhanced by a crosslinking reaction of TDI and rearrangement of the molecule chain. The order and packed structure of the surface is more difficult to etch with the 85% H_2_SO_4_ acid solution.

### 3.2. Mechanical Properties of the Kevlar 49 Fibers

**Figure 5:**
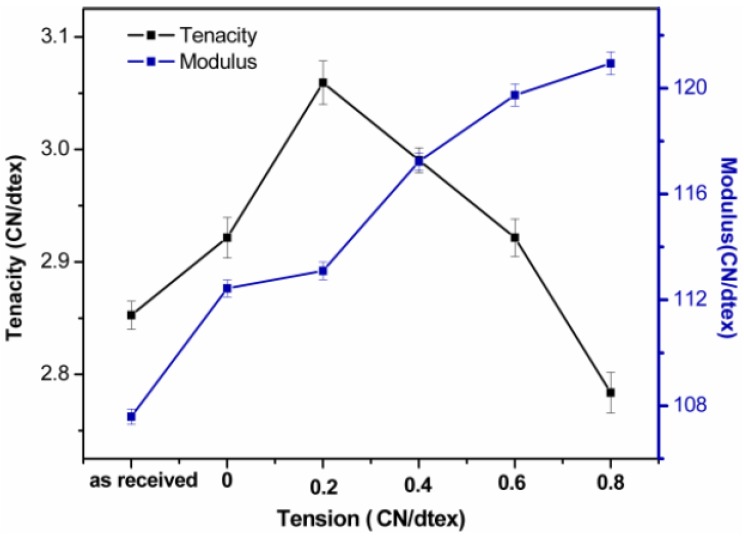
Tenacity and modulus of fibers under various tensions.

The mechanical properties of Kevlar fibers treated under various tensions were studied. The change of modulus and tenacity were shown in Figure 5. Both the tenacity and modulus of fibers were improved. Especially the tenacity increases from 2.85 to 3.05 Gpa, while tensile modulus increases from 107.59 to 120.94 Gpa. Compared to the untreated fibers, the modulus and tenacity increase by nearly 12.4% and 7.7%, respectively. The tenacity was firstly improved when the treating tension was less than 0.6 cN/dtex, but when the tension was larger than 0.6 cN/dtex, the tenacity was decreased. The modulus is significantly improved with the increasing of the tension in scCO_2_ with TDI, the optimal condition for making high modulus is under tension of 0.6 cN/dtex and when we want to obtain a high tenacity, the best condition is 0.2 cN/dtex. Here, TDI was the crosslinker and a solute in scCO_2_. It infiltrated into the fiber, and adhered to the fibrillar elements, thus providing a secondary force for improving the mechanical properties. Moreover, the tensile modulus obviously improves because the molecular chains tend to arrange with the help of the plasticization of scCO_2_ and tension. However, when the tension is too high, owing to the constant pressure and temperature, the tenacity is not obviously improved, especially showed decrease under 0.8 cN/dtex.

### 3.3. Crystal Structure of a Kevlar 49 Fiber in scCO_2_ from the WAXS Results

**Figure 6:**
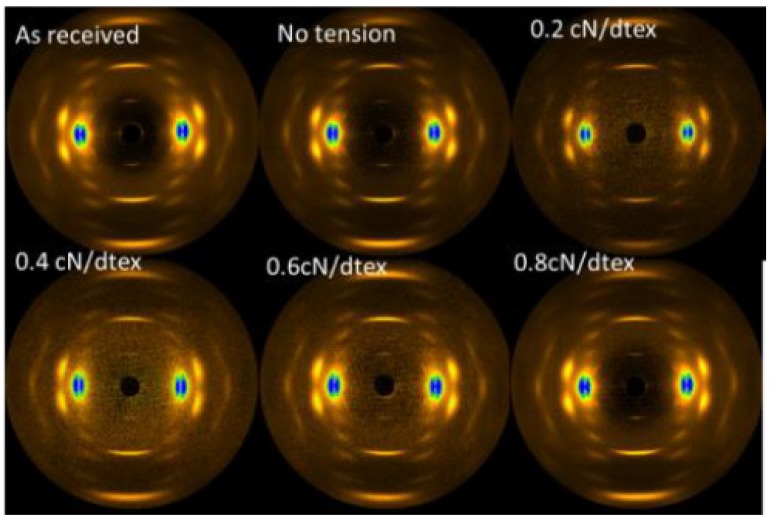
WAXS patterns of Kevlar 49 fibers under various tensions.

**Figure 7:**
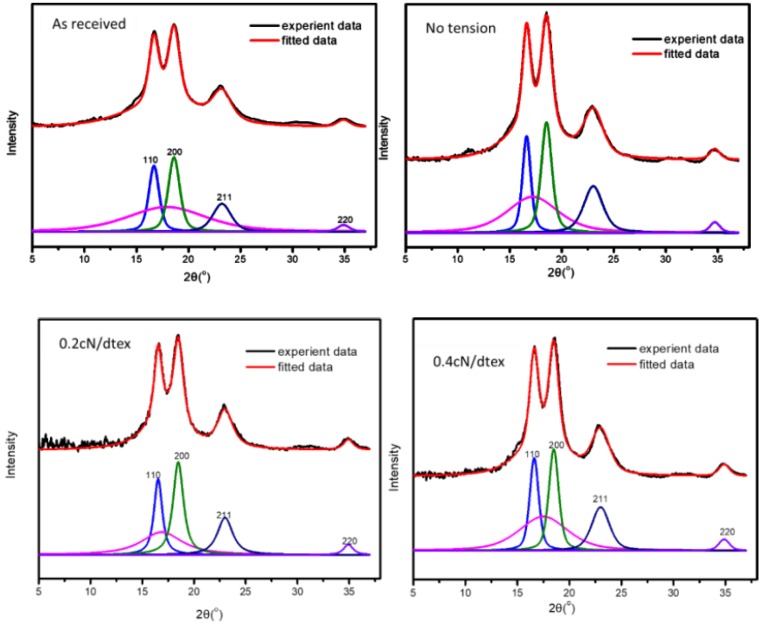

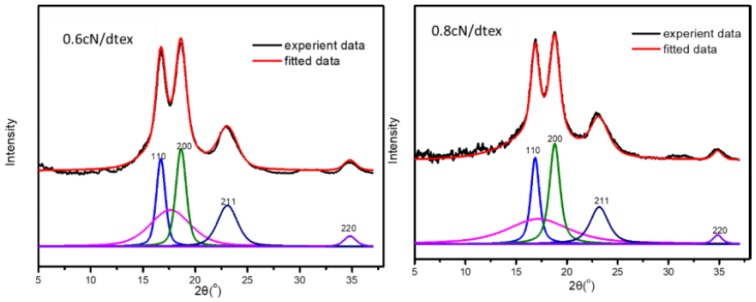
WAXD peaks fitting results for the Kevlar 49 fibers treated in different conditions.

The influence of the treatment tension in scCO_2_ on the crystal structure and orientation is studied by WAXS. The 2D WAXS patterns and fitting results of Kevlar 49 fibers treated under various tensions are shown in Figure 6 and Figure 7. These WAXS patterns are very similar to each other, indicating that no new crystal structure is formed.

Obviously, the reflections of the WAXS patterns of Kevlar 49 fibers treated in scCO_2_ under tension of 0.6 cN/dtex exhibit narrow equatorial arcs, which imply a higher degree of crystal orientation. The results suggest that at a high temperature and high pressure, tension would promote some strain-induced crystallization, which is responsible for the increased crystallinity and orientation.

**Figure 8:**
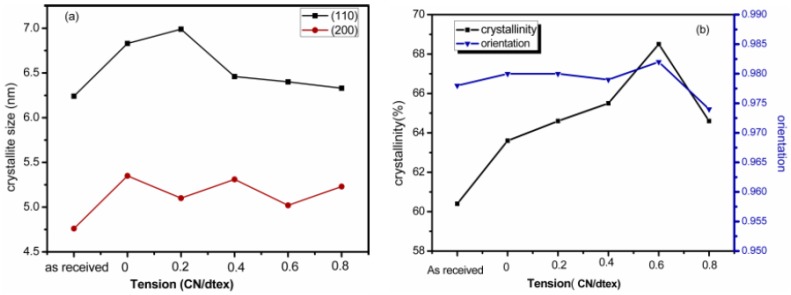
Crystallite sizes (**a**); crystallinity and crystal orientation (**b**) of Kevlar 49 fibers treatment in scCO_2_ under various tensions.

The crystallinity of fibers, crystalline orientation and apparent crystal size of the (110) and (200) peaks of the Kevlar 49 fibers are shown in Figure 8a,b. It is evident that the crystallinity of the fibers after treatment in scCO_2_ mostly increases with increasing tension, indicating that the crystallites region is perfected. The crystallite sizes of the (110) and (200) planes of fibers increase with tension. However, this does not depend on the tension. A tension less than 0.2 cN/dtex, the size of (110) is increased, which indicated that the crystals growing along this direction were increased under tension. However, as some crystals grow with the reorganization in scCO_2_, some crystals might be destroyed at a high tension. The increase in the crystal size also indicates the perfection of the crystallites as the crystals grow.

With the rearrangement of the molecular structure with the plasticization of scCO_2_ and tension in the amorphous region, the perfection of the crystallization was improved. However, for a treatment at a higher tension than 0.6 cN/dtex, the crystallinity slightly increases, but the crystal size and orientation are not greatly increased. This can be explained as follows. When TDI is added, it impregnates into the defect regions in the interior crystal structures, which disrupts the crystal perfection, and the molecules also infiltrate the amorphous regions for crosslinking reactions. These reactions occur between the molecular chains or microfibrils, forming more networks and increasing the intermolecular force, leading to a decrease in the activity of a chain, which may also inhibit its orientation.

As we know, the perfection and orientation of the crystallites are responsible for their mechanical properties [33]. The modulus of the fibers in Figure 5 is consistent with the crystallinity and orientation as shown in Figure 8. Clearly, the increased crystal perfection under tension in scCO_2_ seems to be the cause for the chain alignment and an increase in the modulus while the number of defects is decreased. Moreover, the increase in the intermolecular force due to crosslinking with TDI could improve the tensile strength. However, the crystallites and orientation at the 0.8 cN/dtex tension was less than at the 0.6 cN/dtex tension, which may be because the higher tension would make fibrils broken and less oriented. The crystallite sizes of the (110) and (210) planes were larger, under a higher tension, which is useful for perfection of the crystallites, so the modulus is larger.

### 3.4. Fibrillar Structure Evolution from SAXS Measurements

**Figure 9:**
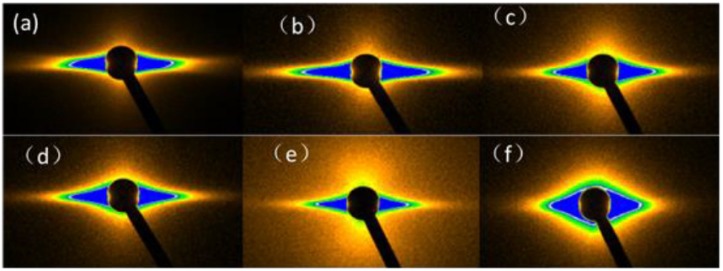
Small-angle X-ray scattering (SAXS) patterns for Kevlar49 fibers treated in different conditions (**a**)—as received, (**b**–**f**)—treated in scCO_2_ with toluene 2,4-diisocyanate (TDI) under various tensions 0, 0.2, 0.4, 0.6, 0.8 cN/dtex.

SAXS was used to determine the effects of TDI on the fibrillar structure of the Kevlar 49 fibers under various conditions. As discussed in Section 2.7, the equatorial streak is related to the microfibrils rather than the microvoids. Figure 9 shows the 2D SAXS patterns of the Kevlar 49 fibers under different tensions. There is a remarkable difference between the untreated and treated fibers in the equatorial direction. The common features in these patterns are that the intensity has a shape of a streak along the equator, and there is no detectable scattering along the meridian direction. On the basis of the SAXS patterns of the Kevlar fibers, the average of length (*L_f_*) and misorientation (*B_obs_*) of a microfibril in the direction of the fiber axis were obtained by the Ruland method.

The degree of orientation and length of the fibril is listed in Table 1. Compared with the as-received fibers, the fibers are treated in scCO_2_ with TDI and without tension, and here are increases in the orientation and correlation length. This result is consistent with the infiltration and crosslinking of a fiber by TDI and the rearrangement of the molecular chains, which will decrease the volume. When the tension is applied, the disorientation of the fibers treated in scCO_2_ decreased under tension by less than 0.6 cN/dex, indicating an increase in the orientation of the microfibrils. However, the length of a microfibril is slightly lower. This reason is that the microfibrils existing between the loosely connected networks of microfibrillar structures are compressed and oriented with the plasticization of scCO_2_. In addition, there is a decrease in the misorientation width of the microfibrils in the fibers treated in scCO_2_ under tension. This may be because the externally applied tensile force will have a stronger effect in the amorphous regions due to their higher mobility since the chains establish a more extended and more densely packed state, decreasing the scattered intensity.

As the 0.6 cN/dtex tension was applied, the microfibrillar length was reduced to 733.4 nm, and the orientation was 11.4°, which is slightly larger than that of the as received fibers and could be attributed to the breakage of some molecule chain under tension, resulting more molecule chain end formed. However, the tensile strength is not decreased largely, as the crosslinking reaction of TDI with chain end happened.

From the tensile properties of the fibers, the fibers treated in scCO_2_ exhibit good mechanical properties. This is because the fibrillar structure plays a significant role in mechanical properties of the Kevlar fibers [39,40]. When the fibers are treated in scCO_2_, they are compressed. Moreover, when TDI impregnates into the amorphous regions of a fiber by scCO_2_, intermolecular crosslinking occurs, and the adhesion between the fibrils increases. The applied tension causes the chains to be arranged more regularly, which would result in crystallization or the perfection of crystallites.

### 3.5. Thermogravimetric Analysis Results

**Figure 10:**
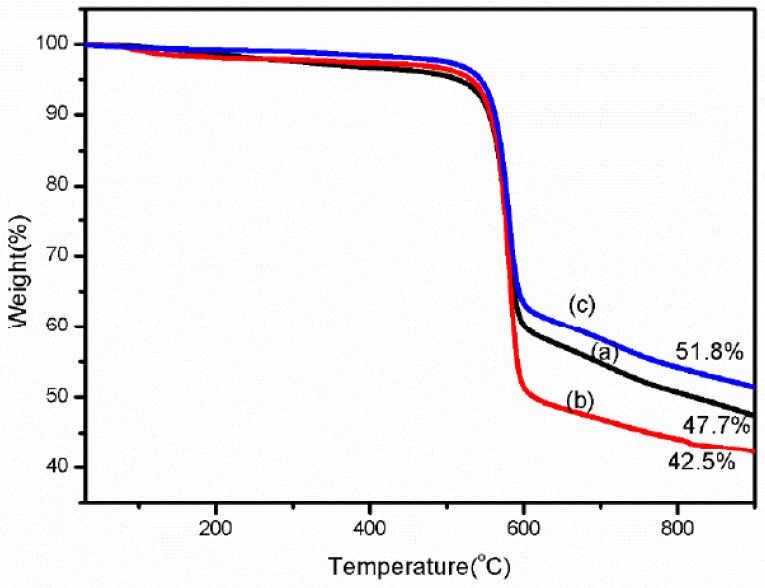
Thermogravimetric analysis (TGA) curves of Kevlar 49 fibers before and after scCO_2_ treatment SAXS Patterns for Kevlar 49 fibers treated in different conditions (a)—as received, (b) treated in scCO_2_ without tension, (c) treated in scCO_2_ under tensions of 0.6 cN/dtex.

We studied the thermal stability of the fibers by thermogravimetric analysis (TGA). From Figure 10, the thermal stability of the Kevlar 49 fiber treated with TDI under a tension of 0.6 cN/dtex (c) is clearly better than the fiber that was only treated in scCO_2_ and without tension (b) and that of the as received fiber (a). From the curves of Figure 9, these fibers show a high stability, as they did not undergo intensive decomposition until 470–580 °C in a nitrogen atmosphere. At this temperature range most likely corresponding to the thermal decomposition of the polymer. Both the thermal stability of the fibers treated in scCO_2_ with or without tension are improved at the 550–580 °C. Moreover, at temperatures above 800 °C, the sample mass loss reaches a nearly constant value which corresponds to the carbon ash content of the sample. The residual mass of the fiber treated in scCO_2_ under a tension of 0.6 cN/dtex is obviously higher than that of untreated fiber and only treated in scCO_2_ without tension. Thermal degradation of fiber starts from skin to core [13], when the fiber treated in scCO_2_ with TDI, the skin surface and amorphous region were enhanced due to the more regular arrangement of the molecules and the higher crystallinity, which corresponding to the results of Figure 4. For the fibers treated in scCO_2_ under tension, the internal crosslinking reaction between TDI and more hydrogen of the amide bond of the fiber, the intermolecular force improves, resulting in an improved heat resistance and residual mass. Moreover, the residual content of the fiber only treated in scCO_2_ is lower, which may be because the impurities or the moisture was reduced by the purification of scCO_2_.

### 3.6. Model of Progress

**Figure 11:**
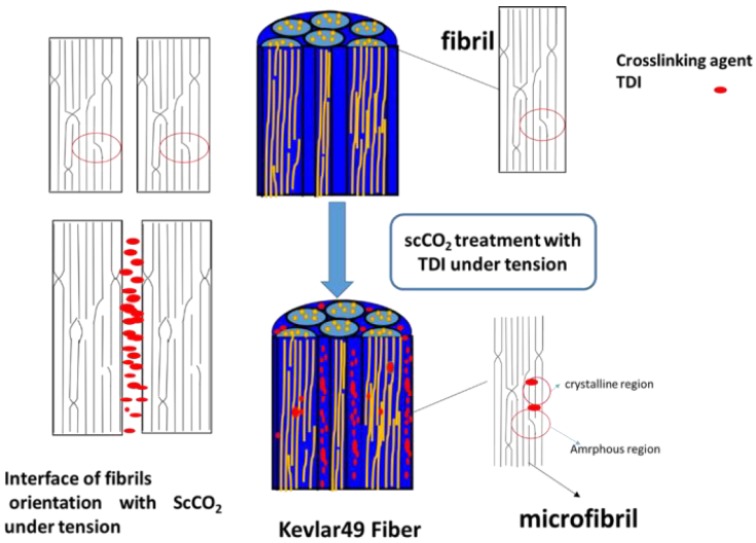
The model of the progress and structure change in scCO_2_ with TDI under tension.

Based on the results and discussions above, a model of the progress and structure change is shown in Figure 11. The Kevlar 49 fiber is comprised of fibrils that are 100 nm^−2^ μm in diameter, which, in turn, are comprised of microfibrils, with a diameter of ~100 nm and length of 500–1200 nm. The microfibril was comprised of a large perfectly oriented crystallite phase and irregular amorphous phase. The amorphous phase has a less regular macromolecule with some chain end or defects, which exist between the crystallites or at fibril interfaces. With the help of swelling the scCO_2_, TDI was dissolved and impregnated the fiber. Crosslinking reaction of TDI with the chain end in the amorphous phase between the interfaces of the fibril will adhere to the fibrillar elements and provide a secondary inter-fibrillar force. With the plasticization of scCO_2_ and tension, the mobility of rigid segment of macromolecular chain is improved, allowing it to be packed more regularly, and the rearrangement of amorphous region, resulting in the perfection of the crystallization with a higher crystallite, orientation, which is useful for improving the mechanical properties.

## 4. Conclusions

Kevlar 49 fibers were treated in scCO_2_ under different tensions to improve the mechanical properties. The effects of the tension of scCO_2_ on the mechanical properties and structure of the Kevlar 49 fibers were studied by mechanical testing, WAXS, SAXS, and SEM. Mechanical testing showed that a suitable tension can simultaneously improve both the strength and modulus of Kevlar49 fiber with the crosslinking reaction of TDI, however, when the tension is higher than 0.6 cN/dtex, the tensile strength is decreased, which may be because the tension that is too high to break some microstructure of the fiber, corresponding to the length of microfibril of SAXS results. As indicated by the results of the measurement of structure from SAXS, the crystallinity of the Kevlar 49 fibers is increased in scCO_2_, owing to the perfection of crystallites and rearrangement in the amorphous regions. With the plasticization of scCO_2_ and tension, the mobility of the rigid segment of macromolecular chain in the amorphous region was improved, and the tension can induce alignment of these rigid segments into a more regular structure. What’s more, the amorphous regions can also be enhanced by the crosslinking reaction of TDI in scCO_2_. The thermal stability of fibers was also be improved as the perfection of structure and improvement of intermolecular force. Owning to the treatment in scCO_2_ can improve both of the tensile strength and modulus of the aramid fiber, it might be applied to the rigid fiber such as polybenzobisoxazole and polyimide fiber. In addition, there appears to be further scope for the research to improve of compressive strength of the rigid fiber.

## Figures and Tables

**Table 1:** The micrfibril parameters of Kevlar49 fibers treated with TDI under different tensions in scCO_2_.

Tension (CN/dtex)	Misorientation Bφ (o)	Microfibril Length L (nm)
as-received	9.7	1224.4
0	6.8	1118.6
0.2	7.9	1150.1
0.4	7.5	863.8
0.6	11.4	733.4
0.8	16.1	936.6
